# Supplementary figures and images for: The species distribution and antimicrobial resistance profiles of Nocardia species in China: A systematic review and meta-analysis
Source: PLoS Negl Trop Dis. 2023 Jul 10;17(7):e0011432. doi: 10.1371/journal.pntd.0011432 (PMC10358964; doi:10.1371/journal.pntd.0011432)

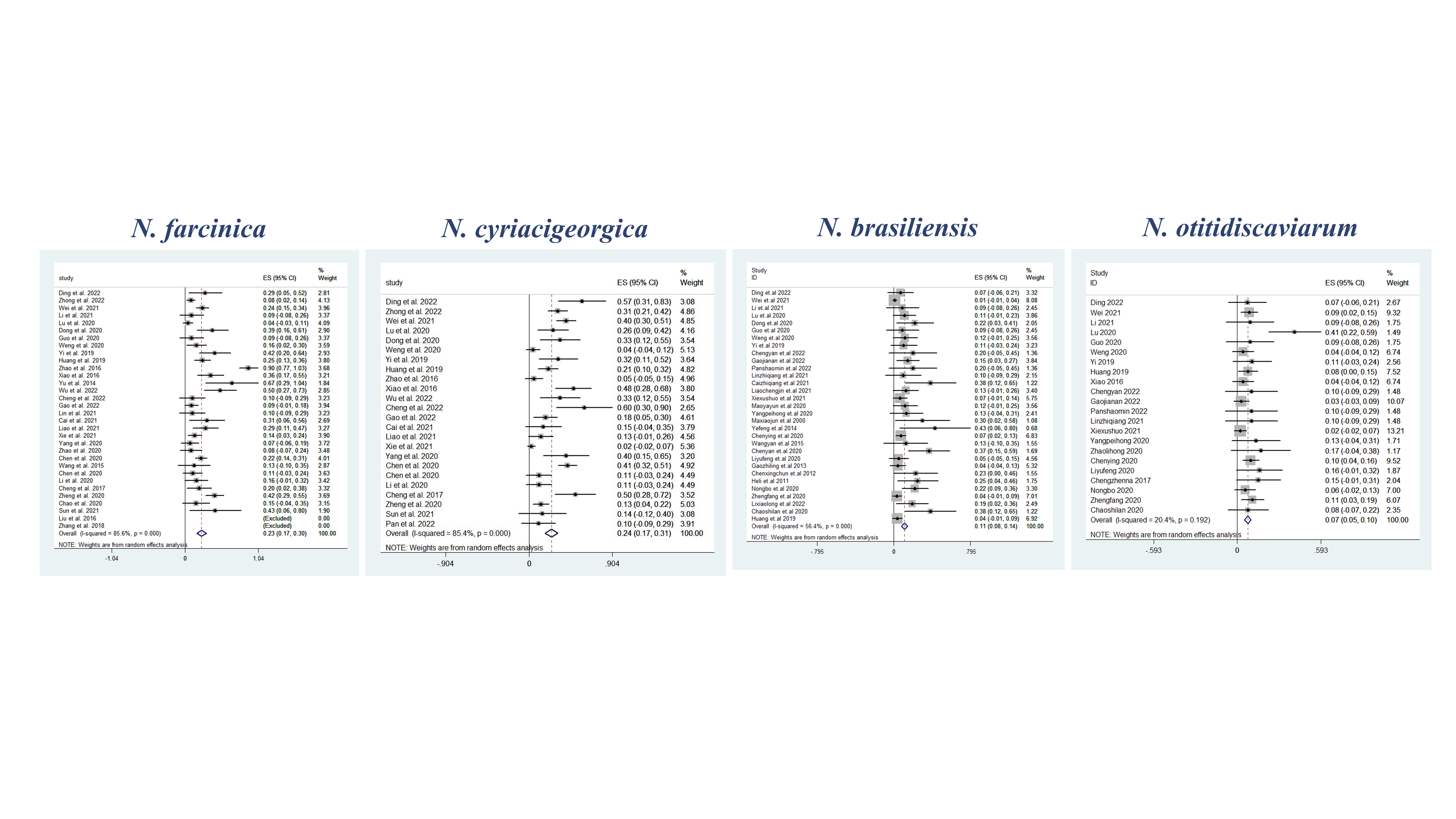

Supplement: S1 Fig — (TIF) [file pntd.0011432.s002.TIF]
