# Supplementary material for: The species distribution and antimicrobial resistance profiles of Nocardia species in China: A systematic review and meta-analysis
Source: PLoS Negl Trop Dis. 2023 Jul 10;17(7):e0011432. doi: 10.1371/journal.pntd.0011432 (PMC10358964; doi:10.1371/journal.pntd.0011432)
Supplement: Attachment — Submitted filename: Review of manuscript PLOS.docx [file pntd.0011432.s003.docx]

Review of manuscript number: PNTD-D-22-01618

The species distribution and antimicrobial resistance profiles of Nocardia species in China: a systematic review and meta-analysis

This manuscript is well written and presents all data in a clear, organized fashion. The table presenting the references for the systematic review displays the method of species ID found in each reference and is very valuable/helpful to the reader.

Please insert line numbers to aid in directing comments to specific text.

In the discussion on the geographic distribution of reported Nocardia species in China, please add a little information about the climate and general ecosystem in each of the regions that have been defined.

In the section on cultural method on Nocardia isolation, it is reported that 80.33% of Nocardia farcinica were isolated by MGIT 960 Myco medium. Were NTM or TB co-infections reported in any of those cases?

In the discussion (and throughout the manuscript) Nocardia asteroides is noted as one of the prevalent species in the southern regions of China. However, reports of Nocardia asteroides *sensu stricto* are quite rare and there is much confusion over the accuracy of any species reported as Nocardia asteroides. Is there assurance that these isolates were in fact accurately identified. Or could this be a misrepresentation due to lack of accurate ID?

Please give a reference for the following statement in the discussion, “Another factor that that may limit Nocardia recovery is that egg-based LJ media is not an optimal choice for Nocardia isolation.”
