# Supplementary material for: The species distribution and antimicrobial resistance profiles of Nocardia species in China: A systematic review and meta-analysis
Source: PLoS Negl Trop Dis. 2023 Jul 10;17(7):e0011432. doi: 10.1371/journal.pntd.0011432 (PMC10358964; doi:10.1371/journal.pntd.0011432)
Supplement: Attachment — Submitted filename: reviewer responses 0518.docx [file pntd.0011432.s004.docx]

**Response Letter**

Dear Editor,

 We thank you and the reviewers for the positive comments regarding our manuscript.

We are grateful to the reviewers for taking time to make the following observations regarding our manuscript, and revised our manuscript as per the reviewers’ recommendations. Here are our responses:

*Reviewer #1: The authors conducted an interesting systemic review and meta-analysis to describe the species distribution and antimicrobial resistance profiles of Nocardia species in China. But this article did not fulfill the spirit of meta-analysis. Meta-analysis is a research process used to systematically synthesise or merge the findings of single, independent studies, using statistical methods to calculate an overall or ‘absolute’ effect. Meta-analysis does not simply pool data from smaller studies to achieve a larger sample size. Analysts use well recognised, systematic methods to account for differences in sample size, variability (heterogeneity) in study approach and findings (treatment effects) and test how sensitive their results are to their own systematic review protocol (study selection and statistical analysis). (*[*https://ebn.bmj.com/content/16/1/3*](https://ebn.bmj.com/content/16/1/3)*) Second, this study did not bring new novelty or new findings to help clinicians.

There were some issues that needed to be addressed: 
1. The manuscript should be checked again for spelling/grammatical mistakes and correct punctuation, e.g. N. farcinica, N. cyriacigeorgica (a space between genus and species), genus/species in italic, gradual increasing (gradually increasing?), Nocardiosis resemble tuberculosis (Nocardiosis resembles tuberculosis?), cinicains (clinicians?), involving 1008 clinical Nocardia isolated (Nocardia isolates?), etc. Make sure that the numbers were correct in regards to those in tables (66.67 %(28/28) N. brasiliensis isolates were susceptible to moxifloxacin??)*

**Reply: Per the suggestion, we checked again for spelling/grammatical mistakes and corrected punctuation.**

*2. A paragraph about the method of paper quality assessment should be added (PRISMA 2020 checklist/QUADAS-2). It should also be included in the result section or supplementary material.*

**Reply: Per the suggestion, we added the method of paper quality assessment using PRISMA 202 in the Method section and presented the result in supplementary material.**

*3. In table 1, the method column and province column should be filled with correct method (e.g. article 4 used PCR for species identification) or cities (multiple provinces should also be stated), not just filled with “-”.*

**Reply: Per the suggestion, we filled with correct method and province in Table 1.**

*4. The distribution of extra-pulmonary specimens should be presented if possible. Different species may cause different types of infection.*

**Reply: Per the suggestion, we added the distribution of extra-pulmonary specimens as following:**

**The skin and soft tissue wounds (41/52, 78.9%) were the main sources of extra-pulmonary infection. *N. brasiliensis* (24/41, 58.5%) and *N. terpenica* (6/41, 14.6%) are the most common species causing cutaneous nocardiosis.**

*5. It may be better to change the sentence “linezolid could be an alternative. Amikacin can be the choice in a combination regimen” to “**linezolid and amikacin could be an alternative to treat nocardiosis or a choice in a combination regimen”.*
**Reply: We changed the sentence as suggestion.**

*Reviewer #2: Comments on "The species distribution and antimicrobial resistance profiles of Nocardia species in China: a systematic review and meta-analysis"

This meta-analysis aims at describing molecular epidemiology at the species level of Nocardia in China. They also analysed antimicrobial resistance profiles.
Overall, the paper is clearly written and the topic is of interest.
However, several major comments need to be taken into account.

Major comments
- Throughout manuscript, the authors report a high proportion of N. asteroides.
It is well documented now that N. asteroides is an exceptional human pathogen. Most of reported N. asteroides have been misidentified. It is therefore very likely that the different proportions of N. asteroides (depending on the cited paper) is rather caused by different proportions of misidentification (depending on the method that is used). As a conclusion, all N. asteroides should be reported as Nocardia spp. and proportions of other species should be recalculated.
This point should be included in the Methods, results and in the discussion section + figures and tables*
**Reply: Thanks for your suggestion. We rewrote the manuscript, reported all *N. asteroides* as *Nocardia* spp. and recalculated the proportions of other species.** *-The authors claim in the Introduction and Discussion sections that "species distribution and antimicrobial resistance profiles remain unavailable for China". As the authors performed a meta-analysis of previously published studies, this statement is, by definition, wrong. Otherwise, metanalysis couls not have been performed. These data exist but the aim of this meta analysis was to bring them together and build a nationwide overview. This should be stated*

**Reply: Per the suggestion, we rewrote the sentence as following:**

**Although increasing attention towards *Nocardia* infection, there is not an organized monitoring system for *Nocardia* spp.. An epidemiological analysis of *Nocardia* spp. mainly depends on meta-analysis or systematic review. This study will build a nationwide overview of species distribution and antimicrobial resistance profiles of *Nocardia* species in China.**
 *- Important bibliographical data are not precise enough, and even wrong.
Introduction: no data support the statement that the incidence of nocardiosis is increasing. The cited paper (REF 2) does not even mention this question.*

**Reply: We admitted that reports are in conflict whether nocardiosis incidence is increasing or remains stable worldwide. So we deleted this sentence.**
 *Introduction and discussion: sentences regarding tuberculosis do not add anything to the paper. This can also be misleading as tuberculosis and nocardiosis are many differences. I would remove these sentences that do not belong to this story.*
**Reply: We admitted that tuberculosis and nocardiosis were different stories. However, *Nocardia* species often isolated among TB suspects in high TB burden settings. In our study, the isolation rate of *N. farcinica* was 80.3% (53/66) by MGIT 960 mycobacterium liquid medium, which was the special medium used for the isolation of *Mycobacterium tuberculosis*. So we briefly described tuberculosis in the Introduction and Discussion section.**

*- Important methodological aspects are lacking
Table 1: the authors mention "PCR" as a "method". What does it mean? Was it the technic for species identification. If so, was it amplification and sequencing or detection? If it was amplification, what was the target gene? What were the interpretation rules (% of base pair similarities)? This should be detailed in Table 1.*

**Reply: Per the suggestion, we described the methods in detail and changed “PCR” to “16S rRNA sequencing” or “16S rRNA and 16S-23S rRNA ITS sequencing” etc. We also added the interpretation rules in Table 1.**

*In the same line, methods for antibiotic susceptilibility testing are not detailed. In this context, it is not possible to compare % of susceptibility.
Was it broth microdilution, E-tests, antibiotic disk diffusion on agar plates?*
**Reply: All the included result about antimicrobial resistance tests was determined using the standard Broth microdilution method, which following the recommendations of the Clinical and Laboratory Standards Institute. Per the suggestion, we described the antibiotic susceptibility testing in detail in Method section.** *Minor comments.
- Throughout manuscript, please add a space after the dot for bacterial names: "N. farcinica" rather that "N.farcinica".*

**Reply: Thanks for your suggestion. We corrected this mistake**

*- For percentage, I suggest limiting to one value after the dot: 70.3% rather than 70.35%.*

**Reply: We changed the percentage as suggestion.**

*- page 14, "causing" rather that "caused"*

**Reply: Thanks for your suggestion. We corrected this mistake.**

*- Discussion: page 19, the sentence regarding trimethoprim/sulfamethoxazole is not accurate. The authors assessed the % of suspectibility but not the "activity". They cannot state that "trimethoprim/sulfamethoxazole still has high-level activity."... They can only state that "trimethoprim/sulfamethoxazole is frequently activite".*

**Reply: We changed the percentage as suggestion.**

*Reviewer #3: This manuscript is well written and presents all data in a clear, organized fashion. The table presenting the references for the systematic review displays the method of species ID found in each reference and is very valuable/helpful to the reader.* 

*Please insert line numbers to aid in directing comments to specific text.* 
**Reply: Thanks for your suggestion. We inserted line numbers.**

*In the discussion on the geographic distribution of reported Nocardia species in China, please add a little information about the climate and general ecosystem in each of the regions that have been defined.*

**Reply: Thanks for your suggestion. We added climate and general ecosystem in each of the regions in the Discussion section.**

*In the section on cultural method on Nocardia isolation, it is reported that 80.33% of Nocardia farcinica were isolated by MGIT 960 Myco medium. Were NTM or TB co-infections reported in any of those cases?*

**Reply: There are 4 studies reported *Nocardia* species isolated from TB suspects using MGIT 960. Only one study showed that among nine patients infected with *N. farcinica*, 5 cases were co-infected with TB. No NTM or TB co-infections were reported in other studies.**

*In the discussion (and throughout the manuscript) Nocardia asteroides is noted as one of the prevalent species in the southern regions of China. However, reports of Nocardia asteroides sensu stricto are quite rare and there is much confusion over the accuracy of any species reported as Nocardia asteroides. Is there assurance that these isolates were in fact accurately identified. Or could this be a misrepresentation due to lack of accurate ID?*

**Reply: Thanks for your suggestion. We rewrote the manuscript, reported all *N. asteroides* as *Nocardia* spp. and recalculated the proportions of other species.** *Please give a reference for the following statement in the discussion, “Another factor that that may limit Nocardia recovery is that egg-based LJ media is not an optimal choice for Nocardia isolation.”*

**Reply: Per the suggestion, we have added the reference.**
